# Viral *E6/E7* oncogene and cellular hexokinase 2 expression in HPV-positive cancer cell lines

**DOI:** 10.18632/oncotarget.22463

**Published:** 2017-11-15

**Authors:** Karin Hoppe-Seyler, Anja Honegger, Felicitas Bossler, Jasmin Sponagel, Julia Bulkescher, Claudia Lohrey, Felix Hoppe-Seyler

**Affiliations:** ^1^ Molecular Therapy of Virus-Associated Cancers (F065), German Cancer Research Center (DKFZ), 69120 Heidelberg, Germany

**Keywords:** human papillomavirus, cervical cancer, hexokinase 2

## Abstract

Oncogenic types of human papillomaviruses (HPVs) are major human carcinogens. Cancer cells typically exhibit metabolic alterations which support their malignant growth. These include an enhanced rate of aerobic glycolysis (‘Warburg effect’) which in cancer cells is often linked to an increased expression of the rate-limiting glycolytic enzyme Hexokinase 2 (HK2). Intriguingly, recent studies indicate that the HPV *E6/E7* oncogenes cause the metabolic reprogramming in HPV-positive cancer cells by directly upregulating HK2 expression. Notably, however, these results were obtained upon ectopic overexpression of E6/E7. Here, we investigated whether HK2 levels are affected by the endogenous E6/E7 amounts present in HPV-positive cancer cell lines. RNA interference analyses reveal that the sustained E6/E7 expression is critical to maintain HK2 expression levels in HeLa cells. Mechanistically, this effect is linked to the E6/E7-dependent upregulation of *HK2*-stimulatory MYC expression and the E6/E7-induced downregulation of the *HK2*-inhibitory micro(mi)RNA miR-143-3p. Importantly, however, a stimulatory effect of E6/E7 on HK2 expression was observed only in HeLa among a panel of 8 different HPV-positive cervical and head and neck cancer cell lines. Thus, whereas these results support the notion that E6/E7 can increase HK2 expression, they argue against the concept that the viral oncogenes, at endogenous expression levels, commonly induce the metabolic switch of HPV-positive cancer cells towards aerobic glycolysis by directly or indirectly stimulating HK2 expression.

## INTRODUCTION

Almost 5% of the total human cancer incidence is closely linked to infections by oncogenic types of human papillomaviruses (HPVs), such as HPV16 and HPV18 [1, 2]. These malignancies include common cancers in the oropharynx and in the anogenital region. The most prevalent HPV-induced malignancy is cervical cancer, which is virtually always caused by oncogenic HPVs and accounts for over 500,000 new cancer cases and over 250,000 cancer deaths worldwide every year [3]. Within cervical cancer cells, the viral DNA is often integrated into the host cell chromosomes [4]. These HPV sequences regularly express the viral *E6/E7* oncogenes which are considered to be crucial for both the induction of HPV-linked malignant cell transformation and the maintenance of the oncogenic phenotype of HPV-positive cancer cells [1, 5, 6].

Cancer cells typically exhibit metabolic alterations which support their malignant growth by promoting cellular proliferation and cell survival [7]. During metabolic reprogramming, cancer cells commonly increase the rate of aerobic glycolysis (‘Warburg effect’) for the generation of energy (ATP) and intermediates for tumor cell growth (e.g. nucleic acid precursors, lipids) [8]. A key regulatory and rate-limiting role in this process is played by the glycolytic enzyme Hexokinase 2 (HK2) which is expressed at only very low concentrations in most normal tissues but often at elevated levels in cancers [9, 10]. Thus, it is of high interest to identify cellular factors that stimulate HK2 expression and thereby induce the metabolic switch towards aerobic glycolysis in cancer cells.

Interestingly, recent studies report a direct link between the activity of the HPV oncogenes and HK2 expression. Specifically, it was shown that ectopic HPV16 E6/E7 overexpression increases HK2 levels and, consequently, glycolysis in mouse embryonal fibroblasts (MEFs) in a MYC (c-myc)-dependent manner [11]. Along this line, ectopic overexpression of E7, but not of E6, also stimulated HK2 expression in cervical cancer cell lines and was linked to an increased proliferation rate and to radioresistance [12]. Based on these results, it was postulated that the *E6/E7* oncogenes reprogram HPV-positive cancer cells towards glycolysis through a direct activation of HK2 expression [11, 12]. This is of high interest for the field, indicating a novel critical activity of the HPV oncogenes for the maintenance of the transformed phenotype of their host cell. Notably, however, *E6/E7* expression levels from the authentic viral promoter are tightly controlled in HPV-positive cancer cells [6, 13] and it is unclear to which extent the observed crosstalk between the viral oncogenes and HK2 expression might be influenced by the intracellular E6/E7 amounts that are generated by the ectopic overexpression from the heterologous promoters used in the studies referenced above.

To address this issue, we here examined a panel of HPV16- or HPV18-positive cervical cancer and head and neck cancer (HNSCC) cell lines which allow to functionally analyze the relation between endogenous E6/E7 and HK2 expression in the background of HPV-transformed cells. In support of the potential of E6/E7 to stimulate HK2 expression, we found that *E6/E7* silencing leads to a strong reduction of HK2 mRNA and protein expression in HeLa cells. Mechanistically, this is mediated, at least in part, by the downregulation of HK2-stimulatory MYC expression and by the upregulation of the HK2-inhibitory micro(mi)RNA miR-143-3p, following *E6/E7* repression. Notably, however, among 8 tested HPV-positive cancer cell lines, *E6/E7* silencing was linked to a decrease of HK2 levels only in HeLa cells. Thus, whereas endogenous E6/E7 can contribute to the maintenance of HK2 levels in HPV-positive cancer cell lines, this appears to be a rare scenario and questions the emerging concept that HK2 levels in HPV-positive cancer cells are commonly dependent on the HPV oncogenes.

## RESULTS

### Relation between HPV E6/E7 and HK2 expression in HPV-positive cancer cell lines

**Figure 1:**
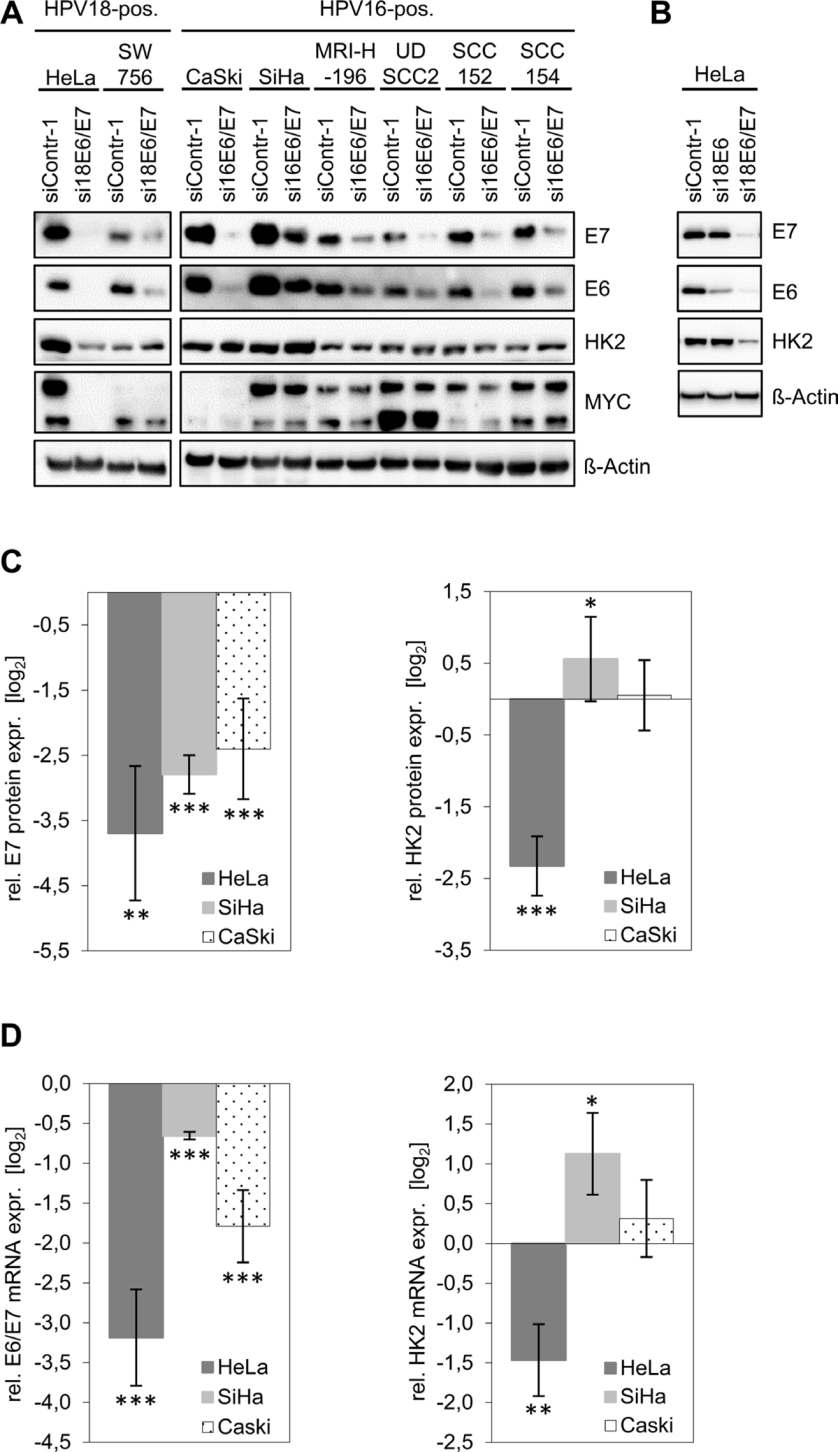
HK2 and MYC levels in HPV-positive cancer cells upon silencing of endogenous *E6/E7* expression (**A**) Immunoblot analyses. HPV *E6/E7* expression was silenced by RNAi in HPV18-positive (HeLa, SW756) and HPV16-positive (CaSki, SiHa, MRIH196) cervical cancer cells and in HPV16-positive (UDSCC2, SCC152, SCC154) HNSCC cells. E7, E6, HK2 and MYC protein levels were determined. siContr-1, control siRNA. β-Actin, loading control. (**B**) Immunoblot analyses. Endogenous HPV18 E6 or E6/E7 expression were silenced in HeLa cells by RNAi. Indicated are E7, E6 and HK2 protein levels. siContr-1, control siRNA. β-Actin, loading control. (**C**) Quantitative analyses of HK2 protein levels upon *E6/E7* silencing. Data is derived from multiple independent experiments in HeLa (*n* = 5), SiHa (*n* = 11) and CaSki (*n* = 6) cells and was quantified by densitometric scanning. For each cell line, relative protein levels are indicated (log_2_ display), compared with control-siRNA-transfected cells. Left panel: E7 protein expression, right panel: HK2 protein expression levels. Standard deviations are depicted. (**D**) Accompanying qRT-PCR analyses of *HK2* transcript levels (right panel) upon *E6/E7* silencing (left panel). Values (log_2_ display) are derived from multiple independent experiments in HeLa (*n* = 5), SiHa (*n* = 11) and CaSki (*n* = 6) cells, and are indicated relative to control-siRNA-transfected cells.

HPV16-positive SiHa, CaSki and MRI-H-196 and HPV18-positive HeLa and SW756 cervical cancer cells as well as HPV16-positive UDSCC2, SCC152 and SCC154 HNSCC cells were transfected with siRNAs that target all three transcript classes coding for HPV16 E6/E7 or HPV18 E6/E7, respectively [14]. Notably, although viral *E6/E7* expression was efficiently reduced by RNA interference (RNAi) in all cell lines, only HeLa cells exhibited a concomitant downregulation of HK2 protein levels (Figure 1A, Supplementary Figure 1). In all other cell lines, HK2 expression remained unchanged (e.g. in CaSki cells) or marginally increased (e.g. in SiHa cells).

Next, we tested whether HK2 expression levels in HeLa cells depend on endogenous HPV E6 or E7 expression. The E6/E7 genes are transcribed in HeLa cells from integrated HPV copies, giving rise to three different transcript classes 1–3 [15, 16]. The specific targeting of class 1 transcripts by RNAi (si18E6) leads to E6 repression [16] (Figure 1B) whereas targeting of mRNA sequences which are common to all three transcript classes (si18E6/E7) leads to the repression of both E6 and E7 (Figure 1B). Despite many efforts, we thus far are unable to selectively silence endogenous E7 expression by RNAi. Clearly, however, whereas E6-specific RNAi leads to a strong downregulation of E6 expression without affecting E7, the HK2 amounts in HeLa cells remained unchanged (Figure 1B). In contrast, combined E6/E7 silencing results in a strong downregulation of HK2 levels. This indicates that the activation of HK2 expression in HeLa cells is linked to the continuous expression of E7, but not of E6, in line with the results of E6 and E7 overexpression in cervical cancer cells [12].

Based on the results of the immunoblot analyses (Figure 1A), we chose HeLa, CaSki and SiHa cells for detailed quantitative analyses of HK2 expression. Investigating HK2 protein levels upon *E6/E7* silencing in 5–11 independent experiments per cell line revealed a strong reduction in HeLa, whereas HK2 expression in SiHa cells slightly increased and remained largely unaffected in CaSki cells (Figure 1C). Accompanying qRT-PCR analyses reveal a corresponding regulation at the *HK2* mRNA level (Figure 1D).

Collectively, these results indicate that HK2 protein and mRNA expression are reduced upon E6/E7 repression only in HeLa cells and not in any of the other investigated HPV-positive cervical cancer and HNSCC cell lines.

### The E6/E7-dependent regulation of HK2 expression in HeLa cells is linked to MYC

**Figure 2:**
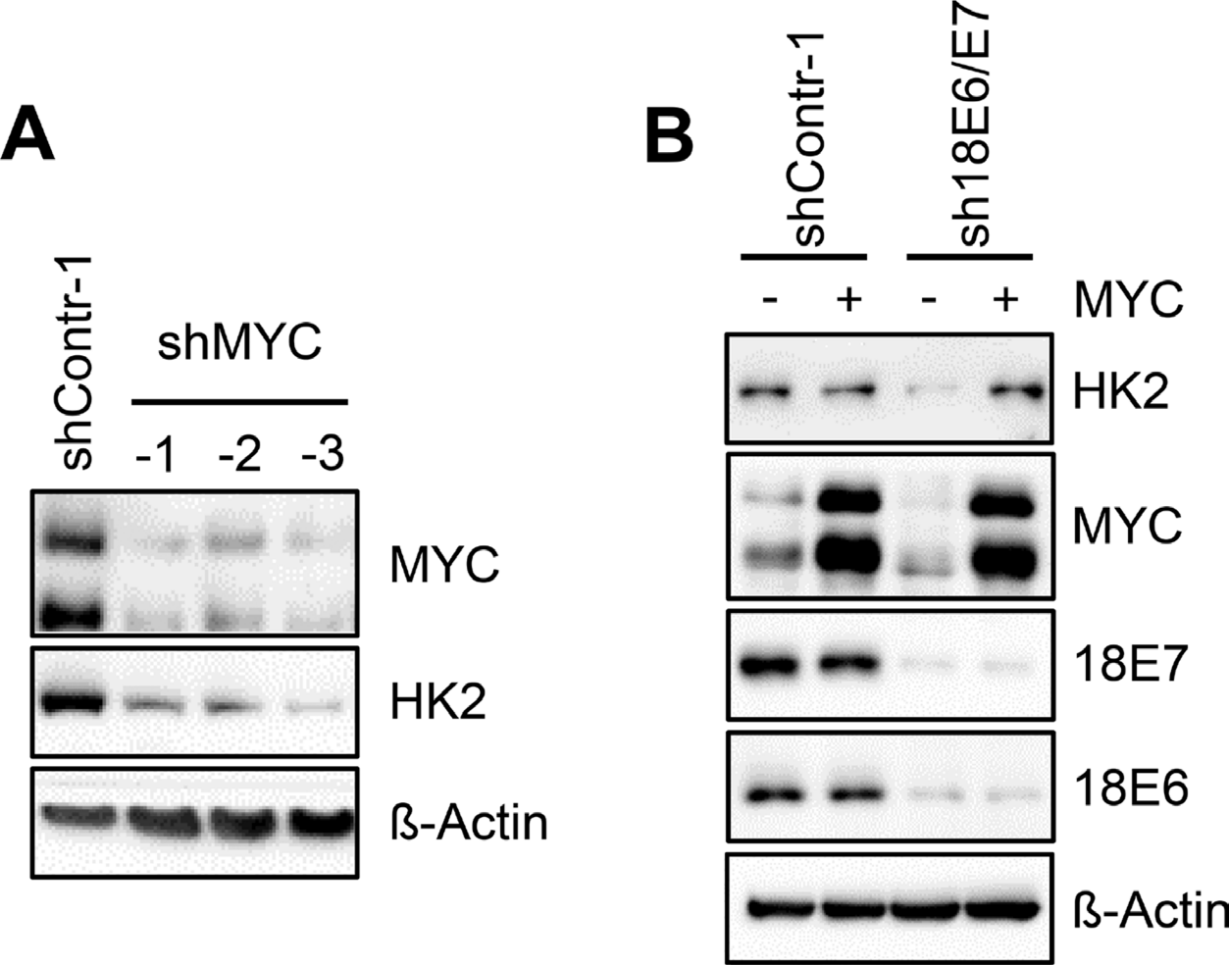
HK2 levels in HeLa cells depend on E6/E7-linked stimulation of MYC expression (**A**) Immunoblot analysis of HeLa cells transfected with three different shRNAs (shMYC-1, shMYC-2, shMYC-3) to block *MYC* expression. Protein levels of MYC and HK2 are indicated. shContr-1, HeLa cells transfected with control shRNA; β-Actin, loading control. (**B**) Immunoblot analysis of HeLa cells transfected with sh18E6/E7 or shContr-1. In addition, cells were concomitantly transfected with a MYC expression vector (+) or the corresponding control vector (–), i.e. the expression vector devoid of *MYC* sequences. Indicated are HK2, MYC and HPV18 E6 and E7 protein levels. β-Actin, loading control.

In line with previous findings at the mRNA level [17], we observed that *E6/E7* repression results in strongly decreased MYC protein expression levels in HeLa cells (Figure 1A). However, like the downregulation of HK2 expression upon *E6/E7* silencing, the profound MYC downregulation was again specific for HeLa cells and was not detected in any of the other investigated HPV-positive cancer cell lines (Figure 1A). Note that the antibody used for MYC detection yields a double band which likely represents phosphorylated and unphosphorylated forms of the protein [18, 19]. Both bands are strongly reduced upon intracellular expression of three different short hairpin (sh)RNAs targeting the *MYC* mRNA, corroborating that the signals are derived from MYC (Figure 2A).

To examine whether the correlation between MYC and HK2 levels in HeLa cells is functionally linked, we tested whether *MYC* silencing leads to the downregulation of HK2 expression. As shown in Figure 2A, shRNA-mediated inhibition of *MYC* expression resulted in decreased HK2 protein levels. This indicates that the E6/E7-dependent HK2 expression in HeLa cells is maintained, at least in part, via the E6/E7-induced expression of MYC. In support of this conclusion, the downregulation of HK2 expression upon *E6/E7* silencing in HeLa cells can be counteracted by ectopic expression of MYC (Figure 2B).

### The E6/E7-mediated regulation of HK2 expression in HeLa cells is linked to miR-143-3p

**Figure 3:**
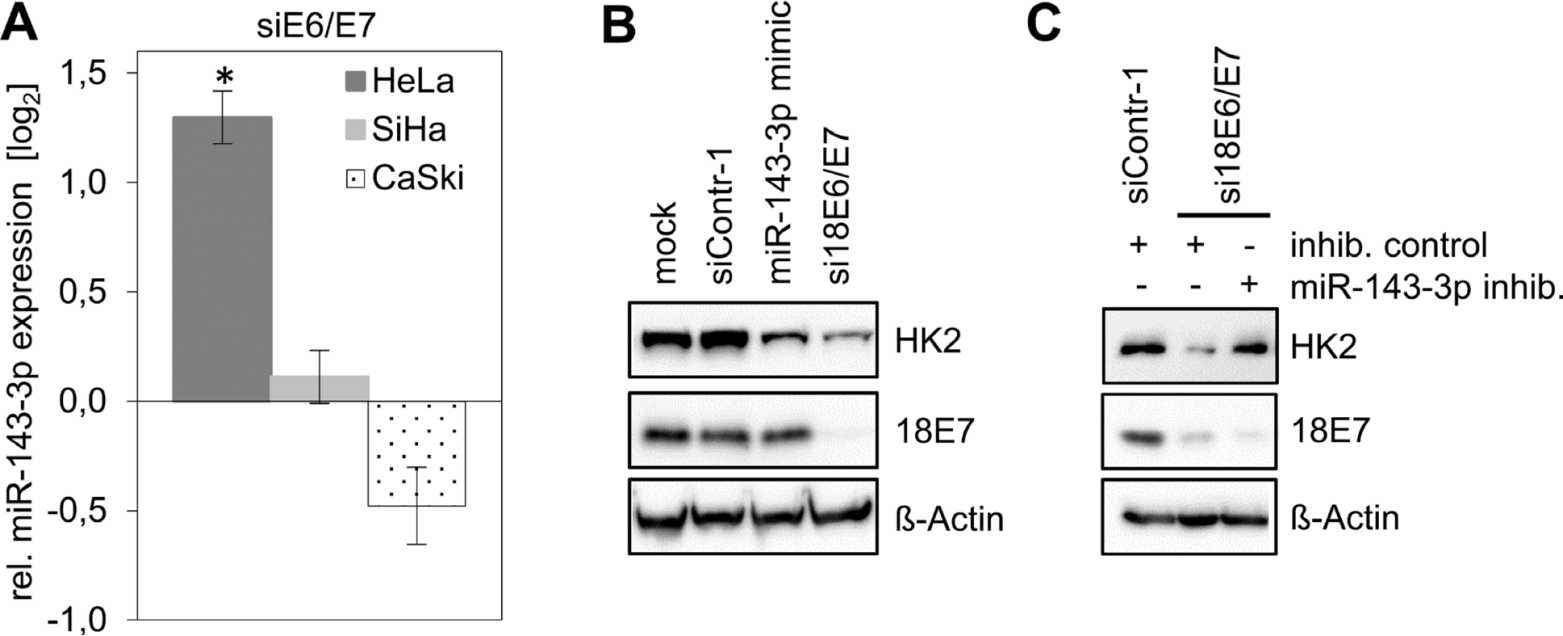
HK2 levels in HeLa cells depend on E6/E7-linked repression of miR-143-3p expression (**A**) Analyses of miR-143-3p levels in HeLa, SiHa and CaSki cells upon silencing of viral *E6/E7* expression by RNAi. Indicated are changes of relative miRNA levels upon transfection of E6/E7-targeting siRNAs (siE6/E7; i.e. si18E6/E7 in HeLa or si16E6/E7 in SiHa and CaSki cells) compared with control-siRNA (siContr-1)-transfected cells (log_2_ display). Data is derived from 3 (HeLa), 4 (SiHa) and 4 (CaSki) independent analyses, respectively. Standard deviations are depicted. (**B**) Immunoblot analysis of HK2 and HPV18 E7 protein levels in HeLa cells following transfection with siContr-1, with a synthetic miR-143-3p mimic or with si18E6/E7, respectively. Mock control, treatment with the transfection agent only. (**C**) Immunoblot analysis of HeLa cells transfected with si18E6/E7 or control siRNA siContr-1. In addition, cells were co-transfected with miR-143-3p inhib. or inhib. control, as indicated. HK2 and HPV18 E7 protein levels are illustrated. β-Actin, loading control.

In a deep sequencing analysis of E6/E7-regulated miRNAs, it was found that–among the most abundant intracellular miRNA species (>1000 reads per million)–miR-143-3p shows the highest upregulation following endogenous E6/E7 repression in HeLa cells [14]. This miRNA is well defined as a negative regulator of *HK2* expression [20–23]. Notably, and in contrast to the situation in HeLa cells, miR-143-3p was not upregulated upon *E6/E7* repression in CaSki or in SiHa cells (Figure 3A). Thus, the ability of HeLa cells to repress HK2 expression upon *E6/E7* silencing correlates with the induction of miR-143-3p, raising the possibility that this miRNA directly contributes to the regulation of HK2 expression by E6/E7.

To investigate this question, we treated HeLa cells with chemically synthesized miR-143-3p mimics and inhibitors. Introduction of a miR-143-3p mimic did not affect HPV E7 protein levels but resulted in a reduction of HK2 protein amounts (Figure 3B), indicating that miR-143-3p is able to repress *HK2* expression in HeLa cells.

Next, we addressed the question whether the miR-143-3p induction upon *E6/E7* silencing (Figure 3A) is involved in the concomitant downregulation of HK2 expression in HeLa cells. In this case, the inhibition of miR-143-3p function should counteract the HK2 repression that is observed upon *E6/E7* silencing. Thus, we blocked E6/E7 expression by siRNA and simultaneously introduced either a control miRNA inhibitor (‘inhib. control’) or a specific miR-143-3p inhibitor. We found that the miR-143-3p inhibitor counteracted HK2 repression in response to *E6/E7* silencing (Figure 3C). Collectively, these data show that HK2 repression upon *E6/E7* silencing is linked to the induction of the *HK2-*targeting miR-143-3p, indicating that the downregulation of this miRNA species by E6/E7 contributes to the elevation of HK2 expression in HeLa cells.

## DISCUSSION

HPV-positive cervical cancer cells are considered to be ‘oncogene addicted’ [24] in that their growth is dependent on the continuous expression of the viral *E6/E7* oncogenes [1, 5, 6]. The definition of the cellular targets attacked by the viral oncogenes is therefore of high interest in order to decipher the critical molecular mechanisms of HPV-linked malignant cell transformation and to identify new pathways for therapeutic intervention [6].

Studies reporting increased HK2 expression and activity in cervical cancers compared to normal cervical epithelium date back to the 1970s [25, 26]. In recent immunohistochemistry analyses, normal cervical tissues exhibited little or no HK2 signals whereas approximately 60% of the cervical cancer specimens (*n* = 197) stained positive for HK2 [11]. This scenario could also be of clinical relevance since increased HK2 expression is a negative prognostic marker for several solid tumors [27]. These include cervical cancer [11, 12, 27] where high HK2 levels are also linked to radiation resistance [12, 28], in line with the concept that increased glycolysis can confer resistance of cancer cells to radiotherapy [29]. As a consequence of its tumor-promoting potential and elevated expression in many tumors, HK2 is discussed to serve as a novel target for cancer therapy [30].

It is assumed that cancer cells benefit from increased HK2 expression by various means. HK2 stimulates aerobic glycolysis which provides energy and the necessary building blocks for tumor cell growth [8]. In addition, HK2 plays an important role for glutamine-derived carbon utilization in anaplerosis, supplying intermediates for the TCA (tricarboxylic acid) cycle [31], and contributes to the maintenance of cellular energy homeostasis by facilitating autophagy in response to glucose deprivation [32]. Moreover, HK2 binds to the pore-forming outer mitochondrial membrane protein VDAC (voltage dependent anion channel) [33]. This interaction is important for the cellular ATP supply by coupling glycolysis to oxidative phosphorylation [34]. Furthermore, the HK2/VDAC interaction can block apoptosis via several mechanisms [10, 35], including by interfering with the formation of the mitochondrial permeability transition pore [36] and by inhibiting pro-apoptotic proteins that target the outer mitochondrial membrane [37]. Thus, the recent reports demonstrating the potential of E6/E7 to stimulate expression of HK2 [11, 12] are intriguing, since they provide a direct connection between the HPV oncogenes and the expression of a key cellular enzyme responsible for the metabolic reprogramming and apoptotic resistance of cancer cells, which is linked to increased oncogenicity and decreased therapeutic sensitivity in the clinic.

However, the studies reporting the stimulation of HK2 expression by the HPV oncogenes were based on ectopic E6/E7 overexpression [11, 12] and it is not clear to which extent the E6/E7 concentrations generated in these experiments reflect the biological effects of the endogenous E6/E7 amounts generated in HPV-positive cancer cells. Here, we found that the endogenous HPV oncogene expression is critical for maintaining HK2 expression in HeLa cells, since *E6/E7* silencing results in a strong downregulation of both HK2 transcript and protein levels. Mechanistically, this response is linked to at least two processes. *First*, *E6/E7* repression in HeLa cells results in a reduction of *MYC* transcript [17] and MYC protein levels (Figure 1A), thereby downregulating a known activator of *HK2* transcription [38]. This mechanism also appears to contribute to the upregulation of HK2 expression upon ectopic overexpression of E6/E7 in MEFs which is linked to MYC [11]. Further, it recently has been reported that HPV E6 can stabilize the MYC protein [39] and thus *E6/E7* repression could additionally lead to an increased MYC protein turnover. *Second*, *E6/E7* repression results in a significant induction of miR-143-3p [14], indicating that E6/E7 downregulates the levels of this abundant *HK2* inhibitory miRNA. Both processes seem to contribute to the stimulation of HK2 expression by E6/E7 in HeLa cells, since HK2 repression upon *E6/E7* silencing could be counteracted by the ectopic expression of MYC as well as by miR-143-3p inhibition. Importantly, however, HK2 repression upon silencing endogenous *E6/E7* expression was only observed in HeLa cells among 8 different tested HPV-positive cervical cancer and HNSCC cell lines.

Another difference between the overexpression studies referenced above and our study is the fact that cervical cancer cells react with a proliferative stop and rapid induction of senescence following silencing of endogenous E6/E7 expression [6]. As was shown for HeLa and SiHa cells under the experimental conditions used of the present study (harvesting the cells 72 h after transfection of E6/E7-inhibitory siRNAs), the cells reconstitute p53 and pRb signaling, are growth arrested, and will be senescent or senescing [40]. Yet, HK2 expression is strongly downregulated by E6/E7 silencing only in HeLa cells, suggesting that the reconstitution of the p53 and pRb pathways or the growth-arrested/senescent phenotype of the cells do not explain the differential crosstalk between E6/E7 and HK2 in HeLa and SiHa cells.

The lack of correlation between E6/E7 and HK2 expression in the context of HPV-positive cancer cells, however, does not preclude that E6/E7 may stimulate HK2 expression at some step of the life cycle of HPVs which is closely linked to the differentiation status of the infected keratinocyte [5]. Notably, other viruses also possess the potential to stimulate HK2 expression, including EBV (Epstein-Barr virus) [41], KSHV (Kaposi’s sarcoma-associated virus) [42], HCV (hepatitis C virus) [43], DENV (Dengue virus) [44] or Ad5 (adenovirus 5) [45]. Similar as observed for endogenous HPV E6/E7 expression in HeLa cells (this study) or for ectopically expressed E6/E7 in MEFs [11], the stimulation of HK2 expression by Ad5 E4ORF1 is linked to the activation of MYC [45]. It is conceivable that viruses profit from stimulating HK2 expression in their life cycle, since enhanced glycolysis provides an increased nucleotide supply necessary for optimal viral replication [44, 45].

The findings of this study also build a basis for future investigations. Firstly, since HeLa cells are derived from an adenocarcinoma whereas all other investigated cell lines stem from squamous cell carcinomas, the question emerges whether the histological background of HPV-positive cancer cells may influence the crosstalk between E6/E7 and HK2. Secondly, MYC is a master regulator of many metabolic processes, including glycolysis, glutaminolysis, nucleotide production or lipid synthesis [46]. The dependency of MYC expression levels on E6/E7 may provide a functional link between the viral oncogenes and these important cancer-linked metabolic circuits in HeLa cells, an issue that deserves further exploration.

Collectively, our results support the notion that the HPV *E6/E7* oncogenes possess the potential to induce HK2 expression. However, HK2 levels in most HPV-positive cancer cell lines are not maintained by the viral oncogenes and the anti-tumorigenic effects observed upon E6/E7 inhibition in these cells are likely not due to decreased HK2 expression. These findings put into question the emerging concept that the *E6/E7* oncogenes commonly maintain the metabolic switch of HPV-positive cancer cells towards aerobic glycolysis by directly or indirectly increasing HK2 expression.

## MATERIALS AND METHODS

### Cell culture, transfections and treatment conditions

HPV18- (HeLa, SW756), HPV16-positive (SiHa, CaSki, MRI-H-196) cervical cancer cells (obtained from the Tumor Bank of the German Cancer Research Center, Heidelberg or from the American Tissue Culture Collection, ATCC) and HPV16-positive HNSCC UDSCC2 [47] and SCC154 [48] cells were cultured in DMEM. HPV16-positive HNSCC SCC152 cells [48] were cultured in MEM containing non-essential amino acids. Media were supplemented with 10% FCS (Gibco, Life Technologies, Carlsbad, CA, USA), 2 mM L-glutamine, 100 U/mL penicillin, and 100 μg/mL streptomycin (Sigma-Aldrich, Saint Louis, MO, USA). The identity of each cell line was verified by multiplex human cell line authentication. Authenticated cells were frozen in aliquots and cells were used in experiments for a maximum of 4 weeks after thawing. Plasmids were transfected by calcium phosphate co-precipitation as described [14]. Expression vector pcDNA3-cmyc was created by Dr. Wafik El-Deiry and obtained from www.addgene.org (plasmid # 16011), pcDNA3.1 vector (Life Technologies) devoid of *MYC*-coding sequences was used as negative control.

siRNAs were either chemically synthesized (Ambion, Life Technologies, Carlsbad, CA, USA) or expressed as shRNAs from vector pSUPER, as previously described [49]. The si/shRNA target sequences were as follows: HPV18 E6/E7-1 5′-CCACAACGUCACACAAUGU-3′; HPV18 E6/E7-2 5′-CAGAGAAACACAAGUAUAA-3′; HPV18 E6/E7-3 5′-UCCAGCAGCUGUUUCUGAA-3′, HPV16 E6/E7-1 5′-CCGGACAGAGCCCAUUACA-3′; HPV16 E6/E7-2 5′-CACCUACAUUGCAUGAAUA-3′; HPV16 E6/E7-3 5′- CAACUGAUCUCUACUGUUA-3′; HPV18 E6-1 5′-GACAUUAUUCAGACUCTGU-3′, HPV18 E6-2 5′-CAGACUCUGUGUAUGGAGA-3′, HPV18 E6-3 5′-CUCUGUGUAUGGAGACACA-3′; MYC-1 5′-CGATGTTGTTTCTGTGGAA-3′; MYC-2 5′-GCTTGTACCTGCAGGATCT-3′; MYC-3 5′-GATGAGGAAGAAATCGATG-3′. Control si/shRNA “Contr-1”, 5′-CAGUCGCGUUUGCGACUGG-3′, contains at least four mismatches to all known human genes.

Synthetic siRNAs (Ambion, Life Technologies) and miR-143-3p mimic (Qiagen, Hilden, Germany) were transfected with DharmaFECT I (Thermo Fisher Scientific, Waltham, MA, USA), according to the manufacturer’s instructions, to reach a final concentration of 10 nM. For silencing of HPV18 or HPV16 *E6/E7* oncogene expression, three different si/shRNAs, which each target all three E6/E7-encoding transcript classes, were pooled at equimolar concentrations to minimize potential off-target effects [14, 40] (referred to in the text as “si/sh18E6/E7” or “si/sh16E6/E7”, respectively). Likewise, HPV18 E6 was silenced by using a pool of equimolar concentrations of three different siRNAs targeting the E6-encoding transcript class 1 [49] (referred to in the text as si18E6). miR-143-3p Inhibitor (Qiagen) and the miScript Inhibitor Negative Control (Qiagen) were transfected with DharmaFECT I (Thermo Fisher Scientific) to reach a final concentration of 100 nM.

In all experiments, cells were harvested for further analyses 72 h after transfection.

### RNA extraction and quantitative reverse transcription-PCR (qRT-PCR)

RNA was isolated with the Pure Link RNA Mini Kit (Ambion, Life Technologies) or, for miRNA detection, with the miRNeasy Mini Kit (Qiagen) following the manufacturer’s protocols. Reverse transcription of 1 μg RNA was carried out with the Proto-Script First Strand cDNA Synthesis Kit (New England Biolabs, Ipswich, MA, USA) or with the miScript II Reverse Transcription Kit (Qiagen) for mRNA or miRNA detection, respectively. qRT-PCR reactions were performed on a 7300 Real-Time PCR System Detector (Applied Biosystems) with the SYBR Green PCR Master Mix (Applied Biosystems, Carlsbad, CA, USA) for mRNA and with the miScript SYBR Green PCR Kit (Qiagen) for miRNA analysis.

The forward (fwd) and reverse (rev) primer sequences (Eurofins MWG, Ebersberg, Germany) were as follows: HPV18 E6/E7 fwd 5?-ATGCATGGACCTAAGGCAAC-3′, HPV18 E6/E7 rev 5′-AGGTCGTCTGCTGAGCTTTC-3′, HPV16 E6/E7 fwd 5′-CAATGTTTCAGGACCCACAGG-3′, HPV16 E6/E7 rev 5′-CTCACGTCGCAGTAACTGTTG-3′; HK2 fwd 5′-GCCCACCTACGTGTGTGCTA-3′, HK2 rev 5′-CACCCCACTTCCCATTCCGA-3′; *ACTB* fwd 5′-AGACAGTATACCCCATGCTGCAT-3′, *ACTB* rev 5′-TCCAATGTGTCTCCATACACAGA-3′; 18S RNA fwd 5′-CATGGCCGTTCTTAGTTGGT-3′, 18S RNA rev 5′-ATGCCAGAGTCTCGTTCGTT-3′. Cycling conditions have been previously described [14]. The sizes of the PCR products were initially analyzed by agarose gel electrophoresis and subsequently checked by melting point analysis after each reaction using the 7300 System SDS Software (Applied Biosystems). Cycle thresholds (Ct) were normalized to the Cts of ACTB or 18S RNA using the comparative Ct (2^–ΔΔCt^) method [50]. Fold enrichments were calculated as compared to the values from the siContr-1 treated samples.

For miRNA analysis samples were run in triplicate for each experiment. Data were analyzed using the comparative Ct (2^–ΔΔCt^) method [50] with the small nuclear RNA RNU6-2 as endogenous control and siContr-1-treated samples as reference. The following miScript Primer Assays (Qiagen) were applied: Hs_miR-143_1 (hsamiR-143-3p), Hs_RNU6–2_1 (RNU6-2).

### Immunoblot analyses

Cellular protein was prepared and analyzed by immunoblotting as described [49]. The following primary antibodies were used: anti-β-actin (A2228; Sigma-Aldrich); anti-HPV18 E7 (E7C); anti-HPV16 E7 (NM2, kind gift of Dr. Martin Müller, German Cancer Research Center, Heidelberg, Germany); anti-HPV18 E6 (AVC 399) and anti-HPV16 E6 (AVC 843) (kind gift from Dr. Johannes Schweizer, Arbor Vita Corporation, Fremont, CA, USA); anti-HK2 (sc-6521; Santa Cruz); anti-c-myc (51-1485GR; BD Pharmingen, San Diego, Ca, USA). Experiments analyzed by immunoblot were repeated at least thrice with consistent results.

Images were monitored using Fusion SL Gel Detection System (Vilber Lourmat, Marne-la-Valleé, France). Band densities were determined by BioID image analysis software (Vilber Lourmat), relative to the respective loading controls.

### Statistical analyses

Fold enrichment data was analyzed following logarithmic transformation. Statistical significance of the data was evaluated by the paired Student’s *t*-test using the Sigma Plot software (Systat Software Inc., San Jose, CA, USA). *P*-values of *p* ≤ 0.05 (*), *p* ≤ 0.01 (**), and *p* ≤ 0.001 (***) were considered statistically significant.

## SUPPLEMENTARY MATERIALS FIGURE


