# Supplementary material for: Viral E6/E7 oncogene and cellular hexokinase 2 expression in HPV-positive cancer cell lines
Source: Oncotarget. 2017 Nov 15;8(63):106342–51. doi: 10.18632/oncotarget.22463 (PMC5739738; doi:10.18632/oncotarget.22463)
Supplement: Supplementary file 1 [file oncotarget-08-106342-s001.pdf]

Viral *E6/E7* Oncogene and cellular hexokinase 2 expression in HPV-positive cancer cell lines

SUPPLEMENTARY MATERIALS

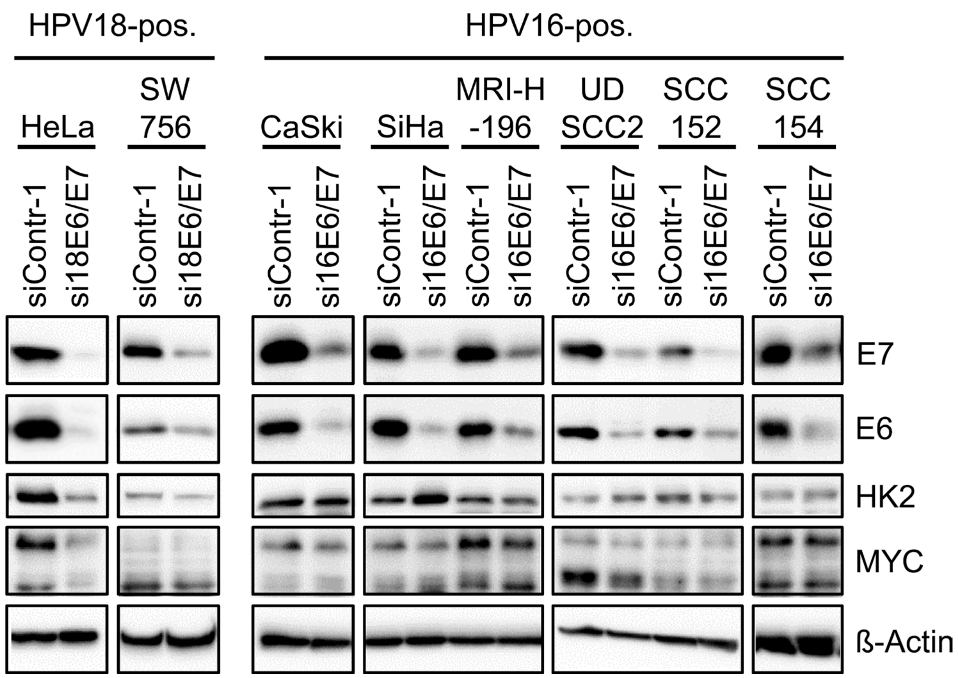

Supplementary Figure 1: HK2 and MYC levels in HPV-positive cancer cells upon silencing of endogenous *E6/E7* expression. Indicated is a biological replicate for each cell line investigated in the immunoblot analyses shown in Figure 1A. For further details, please refer to the legend of Figure 1A.
